# The hidden drivers: Unraveling the impact of density, moisture, and scale on *Hermetia illucens* rearing

**DOI:** 10.1371/journal.pone.0317049

**Published:** 2025-01-08

**Authors:** Anjani Nayak, Patrick Klüber

**Affiliations:** 1 Institute of Food Chemistry and Food Biotechnology, Justus Liebig University Giessen, Giessen, Germany; 2 Fraunhofer Institute for Molecular Biology and Applied Ecology, Giessen, Germany; Universita degli Studi della Basilicata, ITALY

## Abstract

The black soldier fly (*Hermetia illucens*) is a saprophagous insect known for bioconverting organic waste, potentially offering environmental benefits, such as contributing to waste reduction and nutrient cycling. The performance of larvae varies significantly with factors substrate moisture, larval density, and scale of production. Three experiments were conducted using a mix of spent mushroom substrate (SMS) and chicken feed (CF). In the first experiment, 250 larvae were reared on 100 g dry matter (DM) feed at moisture levels of 65–75%. Results showed that the average individual larval weight, total biomass, and feed conversion ratio (FCR) improved with increased moisture. In the second experiment, 300 and 350 larvae/box were tested at 70% and 75% moisture. The highest average individual larval fresh weight (158.6 mg) was observed at 70% moisture with 250 larvae, while the highest biomass was achieved at 75% moisture with 300 larvae. Finally, different scales (10–2,500 g feed with 25–6,500 larvae) were tested with a similar feeding rate. The highest individual larval weight was recorded at the 100 g scale, with no clear correlation between weight and scale. However, the 50 g scale achieved the highest substrate reduction (33.2%). Overall, this study underscores the need to adjust moisture, density, and scale to nutrient conversion efficiency when using SMS, CF or other diets. The optimal results for the SMS feed mix were observed at 75% substrate moisture, 250 larvae per 100 g DM, and at approximately 2 larvae per cm^2^.

## Introduction

The popularity of *Hermetia illucens*, the black soldier fly (BSF; Diptera: Stratiomyidae), has surged significantly in both academic and commercial sectors over the years [1]. The initial studies were mainly based on the larval bioconversion ability [2] and larvae as an alternative feed ingredient for various animals [3]. Recently, the trend has expanded to explore other potential applications, such as using BSF larvae for cosmetics, biodiesel, and various biotechnological products [4–9]. This underscores the importance of sustainable BSF larvae production to meet the growing demand driven by its multisectoral use and popularity [10–13]. Despite this, the knowledge on sustainably optimized feed and rearing methods is limited. The possible reasons include the vast array of plant and animal-based feed options [14, 15], differences in their nutritional composition [16] and physical properties [17], as well as variations in experimental methodologies. The side streams such as fruit and vegetable wastes [18, 19], or other organic wastes [15, 20] are well investigated. However, side streams from the edible mushroom production, although promising, are less explored as feed for BSF larvae.

The production of edible mushrooms increased more than 30 times in less than 50 years globally [21]. China is the world’s largest mushroom producer, responsible for approximately 80% of global output [21]. Additionally, over 100 other countries are involved in mushroom cultivation [22]. This is unsurprising, as materials needed for mushroom cultivation are widely available around the world. Mushrooms are grown on organic substrates like crop residues, wood chips, sawdust, and husks, among others. After mushroom harvesting, organic materials containing residual fungal mycelium, referred to as spent mushroom substrates (SMS), are often discarded [23]. The increasing demand for mushrooms has led to a significant production of SMS [24, 25]. The composition of SMS varies depending on the initial materials used in cultivation. Typically, it consists of cellulose, hemicellulose, lignin, residual fungal mycelium, carbohydrates, proteins, and minerals [26]. The abundant availability of SMS and its potential use as feed for BSF larvae make it an ideal resource for further investigation. Two mushroom species, namely king oyster (*Pleurotus eryngii*) and shiitake (*Lentinula edodes*), have been used as feed for BSF larvae by Li et al. (2021) [27]. However, the number of studies on the use of SMS and its influence on larval performance is limited. Moreover, the influence of factors such as moisture, larval density, and scale are rarely explored.

BSF larvae are known to exhibit varying effects on their survival, growth, feed conversion ratio, and substrate reduction with different substrate moisture levels [17, 28–30]. For example, among the moisture contents of 70–80%, larval growth showed a positive correlation with increasing moisture [31]. A similar result was reported for larval yield by Palma et al. (2018) within a moisture content range of 48–68% wet basis [30]. According to Fatchurochim et al. (1989), BSF larvae can grow and develop into pupae in substrates with moisture content between 30% and 70% by weight [32]. However, the physicochemical characteristics of the substrate determine whether it is too dry, too wet, or optimal for larval growth at a given moisture range. Hence, it is necessary to examine whether the moisture content has to be specifically adapted depending on the diet and its physicochemical properties [33]. The simplest approach is to experiment with different moisture levels for the same substrate and observe the larval performance.

Density plays a crucial role in BSF feeding trial, as it can promote intraspecific competition [34], due to larval aggregation [35] or increase microbial load, leading to better nutrient uptake [36, 37]. Various ranges of BSF larval density have been considered in experiments: Parra-Paz et al. (2015) assessed densities of 1–6 larvae/cm^2^ [5], Opare et al. (2022) tested 1–10 larvae/cm^2^ [38], while Barragan-Fonseca et al. (2018) examined lower densities of 0.3–2.5 larvae/cm^2^ [36]. Given the importance of larval biomass yield and developmental time in industrial-scale productions, further research on density optimization using sustainable feeds is essential.

Various studies have been conducted at different scales. The scale of an experiment refers to the size of the study in terms of the amount of feed and the number of larvae used. In BSF research, the impact of scale is not extensively studied [39]. Most BSF feed trials are conducted on a lab or bench-top scale [40, 41], which is advantageous for fast and resource-efficient testing of larval performance [42]. However, understanding how factors change during scaling up is essential for expanding BSF production using by-products. Additionally, some studies have conducted just the large-scale feed trials without comparing them to smaller-scale processes [39, 41, 43]. A large-scale experiment was carried out using up to 1400 kg FM feed with a larval density of 10 larvae/cm^2^, however no comparisons were conducted based on scale [43]. Yakti et al. (2022) used four box sizes (scales) with low or high densities of larvae each [42]. The number of larvae in the smallest box with low density included 814 larvae (4.2 larvae/cm^2^) and the largest box with high density consisted of 13,000 larvae (6.3 larvae/cm^2^). Here, the authors declared that the scale of the experiment is known to alter the larval composition [42]. There has been no scale experiment using SMS as feed for BSF larvae so far. If the scale experiments in laboratory setup yields different results, further optimization and tailoring will likely be necessary before establishing an optimized production methodology for industrial replication [40, 41].

Taking all these factors into account, three research questions were formulated:

1. How does the moisture content influence larval performance on an SMS-based diet?
2. Does increasing larval density (larvae/cm^2^) improve larval growth performance and feed conversion at substrate moisture levels above 60%?

1. What is the impact of varying experimental scales on the growth performance and survival rates of larvae in a laboratory setup?

## Materials and methods

The experiments were carried out at the Fraunhofer Institute for Molecular Biology and Applied Ecology (Giessen, Germany) between July 2022 and August 2023.

### Insects and feeding substrates

Eight-day-old BSF larvae, vacuum-packed and shipped by Hermetia Baruth GmbH (Baruth/Mark, Germany), were used for the feeding trials. Upon arrival, the larvae were unpacked, transferred into polypropylene boxes, and placed in a climate chamber at 27 ± 1°C and 65 ± 5% relative humidity for 1 h to allow them to recover their natural behavior. For an assured uniform size and mass, the larvae were separated using a vibratory sieve shaker with a mesh size of 1.0–1.4 mm (AS 200, Retsch, Haan, Germany). The average individual larval weight was then determined by weighing five replicates of 100 larvae each using a precision balance (ALJ 160-4A, Kern & Sohn, Balingen-Frommern, Germany). For experiments with less than100 larvae, they were counted individually. BSF larvae that were used for feeding trials had an average individual weight of 7.0 ± 0.0 mg.

The diet consisted of equal parts chicken feed (CF; GoldDott Eierglück, DERBY Spezialfutter, Muenster, Germany) and SMS. The SMS was sourced from an organic mushroom farm (Löckes Bio-Vertriebs GmbH, Büttelborn, Germany). It was collected as a by-product from the production of king oyster (*Pleurotus eryngii*) and shiitake (*Lentinula edodes*) mushrooms on the same day the fruiting bodies were harvested. Initially, any remaining fruiting bodies on the surface of the SMS blocks were removed. The blocks were then manually disintegrated and immediately dried at 80°C for 10 h using a laboratory kitchen oven (HB674GBS1, Siemens AG, Munich, Germany). The CF and dried SMS were processed to a particle size of 0.1–0.8 mm using a Mockmill 200 (Wolfgang Mock, Otzberg, Germany) and a Thermomix TM6 (Vorwerk, Wuppertal, Germany). The prepared substrates were stored in airtight containers at room temperature until further use. For the feeding trials, king oyster SMS and shiitake SMS were combined in a 1:1 ratio and are hereafter referred to as SMS.

### Optimization of substrate moisture

The dry matter (DM) weight of each diet, amounting to 100 g, was distributed into three replicate cylindrical boxes measuring 12.5 cm diameter and 11.4 cm height each (BDPN24, MegaView Science, Taichung, Taiwan). The dry matter content of the substrates was determined thermogravimetrically using a moisture balance (DAB 100–3, Kern & Sohn, Balingen-Frommern, Germany). Moisture levels of 65%, 70%, and 75% were achieved by adding warm tap water. Following this, 250 eight-day-old BSF larvae were evenly distributed over the substrate surface in each box (2.04 larvae/cm^2^). A circular 9 cm mesh insert was placed in the donut-shaped lid to ensure adequate air circulation. All boxes were then placed in a climate chamber under controlled conditions of 27 ± 1°C and 65 ± 5% relative humidity in darkness. The boxes were randomly repositioned every second day. No additional feed or water was added throughout the experiment. Following the outcomes of preliminary experiments, larvae from all moisture groups were harvested ten days after the feeding commenced. Each larva was individually retrieved from the frass using spring steel tweezers, cleaned to remove coarse impurities, weighed, counted, and then cold-inactivated at -20°C. The survival rate was calculated as the percentage of larvae, prepupae, and/or pupae recorded during the harvesting process in comparison to the initially inserted larval numbers. The total harvested biomass denotes the total amount of insect biomass collected per box, comprising larvae, prepupae, and pupae, minus the initially inserted larval weight. Based on this, the average individual larval weight was determined by dividing the total harvested biomass by the number of surviving larvae, expressed as fresh matter (FM). The feed conversion ratio **(Eq 1)** was calculated based on DM [44]:

$$FCR\;(Feed\;conversion\;ratio)=\frac{Total\;feed\;provided\;(g)}{\;(Total\;biomass\;harvested\;(g)-Initial\;biomass\;inoculated\;(g))}$$ (1)

### Density optimization for selected moisture contents

Higher moisture contents (70% and 75%) showed improved larval growth, prompting a retest with increased larval densities (300 and 350 larvae per box i.e., 2.45 and 2.85 larvae/cm^2^, respectively) to evaluate survival and growth performance. This experiment was carried out under the same conditions as for the moisture optimization. The objective was to investigate whether and how the variables moisture and density interact. Feed and larval samples were stored at -20°C for subsequent analysis. Data collection and calculations followed the previously mentioned methodology.

### Investigation of scale effects on growth performance and bioconversion

After optimizing density and substrate moisture, the focus was on determining the extent to which the scale of the feeding trials has an influence on larval growth and bioconversion efficiency. Therefore, at first, it was intended to clarify how the survivability and the growth performance of larvae are affected if the same experiments are done in different scales. A total of five scales were examined (10 g feed + 25 larvae, 50 g feed + 125 larvae, 100 g feed + 250 larvae, 1,000 g feed + 2,500 larvae, 2,500 g feed + 6,500 larvae), with 75% substrate moisture. The diet used was 50% CF and 50% SMS. The feeding rate was the same in all the setups i.e., 0.4 g DM/larva.

For the smaller scale setups (10 g, 50 g, 100 g DM feed), the corresponding amount of each diet was weighed (ALJ 160-4A, Kern & Sohn, Balingen-Frommern, Germany) into three cylindrical boxes with 12.5 cm diameter and 11.4 cm height replicate boxes (BDPN24, MegaView Science, Taichung, Taiwan), as described in the previous chapters. A circular 9 cm mesh insert was placed in the donut-shaped lid to ensure adequate air circulation in the smaller scale boxes. The substrate moisture was adjusted to 75% by adding warm tap water. Subsequently, 25 and 125 eight-day-old BSF larvae were counted individually and transferred in each box by spreading them over the substrate surface. For other scales, the average weight of individual larvae was calculated by weighing five replicates of 100 larvae each using a precision balance (ALJ 160-4A, Kern & Sohn, Balingen-Frommern, Germany). The substrate was weighed using the same balance for the 10 g, 50 g, and 100 g DM feed approaches. Because of the larger amount of feed, the 1,000 g DM (Kern 572–39, Kern & Sohn, Balingen-Frommern, Germany) and 2,500 g DM (ACS-Z, CELMI, Buccinasco, Italy) feed were weighed using different balances and transferred into three cuboidal replicate boxes of 17.0 × 25.5 × 38.0 cm (Santos Box XS, Keter Italia S.p.A., Roncadelle, Italy) and 39.4 × 29.4 × 24.7 cm (AUER packaging, Amerang, Germany), respectively. A 1,000 mesh/6.5 cm^2^ cloth (A113a, Bioform, Nuremberg, Germany) was used for a proper air circulation and to prevent larval escape for 1,000 g and 2,500 g feed boxes. The number of larvae per cm^2^ were 0.20, 1.02, 2.03, 5.77, and 5.61 for the feed 10–2,500 g, respectively. The variations in the density per cm^2^ is due to the different box sizes. All boxes were incubated in a climate chamber under identical conditions as in previous experiments. The boxes were rearranged randomly on every second day. No additional feed or water was added throughout the experiment and boxes were harvested 10 d after the trial has started. The harvesting procedure and storage protocols mirrored those applied in the previous experiments. Likewise, survival rate, total biomass, average individual larval weight, and FCR were calculated following the same methods. The FCR measures the relationship between the quantity of feed provided and the weight gained by the larvae. In addition, frass was collected, weighed and the DM content determined. The efficiency of conversion of ingested feed (ECI), substrate reduction (SR), and waste reduction index (WRI) were calculated following Eqs (2–4) [44, 45]. The equations ECI and FCR assume that larvae consume all the provided feed, unlike conventional livestock, where feed intake can be measured accurately. The factors total harvested biomass (DM and FM) and frass weight were normalized based on the number of larvae to allow comparability between the different scales.


$$ECI\;(Efficiency\;of\;conversion\;of\;the\;ingested\;feed)=\frac{\;(Total\;biomass\;harvested\;(g)-Initial\;biomass\;inoculated\;(g))}{\;(Total\;feed\;provided\;(g)-Frass\;residue\;(g))}$$ (2)

$$SR\;(Substrate\;reduction\;(\%))=\frac{\;(Total\;feed\;provided\;(g)-Frass\;residue\;(g))\times 100}{Total\;feed\;provided\;(g)}$$ (3)

$$WRI\;(Waste\;reduction\;index)=\frac{\;(Total\;feed\;provided\;(g)-Frass\;residue\;(g))}{Number\;of\;rearing\;days\;(d)}$$ (4)

### Data processing and statistics

Data curation and processing were carried out using Excel 2016 (Microsoft, Redmond, WA, USA). Statistical analysis and visualization were conducted in OriginPro 2024b (OriginLab, Northampton, MA, USA). The Shapiro–Wilk test was applied to verify whether the data of a population is normally distributed. The homogeneity of variance was calculated with Levene’s test. Variables recorded in the moisture and scale experiments were subjected to a one-way ANOVA and means were separated using the Tukey’s test (homogeneous variance). If the variance was inhomogeneous, a one-way Welch’s ANOVA followed by Games–Howell post hoc test was conducted. The Pearson product–moment correlation was used for determining linear relationships between variables [46]. Data obtained from the density experiment were subjected to a two-way ANOVA and means were separated using the Tukey’s test. An error level of *α* = 0.05 for statistical significance was set for all analyses. For the scale experiment, total harvested biomass, frass weight and WRI were normalized based on the corresponding number of larvae.

## Results

### Optimization of substrate moisture

The first trial examined how varying substrate moisture content affects larval growth and development (Table 1). The goal was to determine the optimal moisture level among 65, 70, and 75% for a substrate composed of 100 g DM feed, with an equal mix of CF and SMS. The survival rate of larvae was ≥ 93% and did not differ significantly between the moisture groups (*P* = 0.12; Table 1). However, a negative linear correlation between the survival rate and the moisture level was calculated (*r =* −0.74; *P =* 0.02). The average individual larval weight (233.3 mg FM) was highest in the 75% moisture level group and increased with rising moisture levels (*r* = 0.99; *P* < 0.01). Here, larvae grew significantly better and reached an average individual weight that was 49.8% and 120.3% higher when compared to the 70% and 65% moisture groups, respectively (*P* < 0.01). Both fresh matter and dry matter of the total harvested biomass were highest in the 75% moisture group, outperforming the other groups by 35.0–133.7% (*P* < 0.01; Table 1). The total harvested biomass was strongly positively correlated with increasing moisture level (*r* = 0.99; P < 0.01). Providing the larvae feed with 75% moisture improved the feed conversion ratio (FCR) by 50.3% in comparison to the 65% moisture group (*P <* 0.01; Table 1). Here, FCR correlated negatively linearly with increasing moisture level (*r* = −0.98*; P <* 0.01).

**Table 1:** Growth performance and feed conversion ratio of BSF larvae reared at different moisture levels (65–75%).

	Substrate moisture (%)		
Variables	65	70	75
**Total amount of feed (g DM)**	100.0 ± 0.0 ^a^	100.0 ± 0.0 ^a^	100.0 ± 0.0 ^a^
**Survival rate (%)**	100.0 ± 0.1 ^a^	97.9 ± 3.0 ^a^	93.2 ± 3.0 ^a^
**Average Individual larval weight (mg FM)**	105.9 ± 1.7 ^a^	158.6 ± 9.0 ^b^	233.3 ± 8.2 ^c^
**Total harvested biomass (g FM)**	26.5 ± 0.7 ^a^	39.3 ± 0.5 ^b^	53.1 ± 1.3 ^c^
**Total harvested biomass (g DM)**	7.9 ± 0.2 ^a^	13.6 ± 0.2 ^b^	18.4 ± 0.4 ^c^
**Feed conversion ratio (FCR)**	3.8 ± 0.1 ^a^	2.5 ± 0.0 ^b^	1.8 ± 0.0 ^c^

Data represent means ± SD (*n* = 3). Different letters (a–c) within a row indicate statistically significant differences between groups (*P* < 0.05; one-way ANOVA or Welch’s ANOVA). DM = dry matter, FM = fresh matter, FCR = feed conversion ratio

### Density optimization for the selected moisture contents

In general, larval performance improved with higher substrate moisture content. Consequently, substrate moisture levels of 70% and 75% were selected for further investigation. Building on the previous experiment, where larval density was set at 250 larvae per 100 g DM, this study explored variations in stocking density ranging from 250 to 350 larvae per box (S2 Table).

All groups received the same amount of 100 g DM feed consisting of CF and SMS (1:1 *w/w*). The survival rate of all groups ranged between 83.7–100.0% with statistically significant differences between the moisture groups (*P* < 0.01). A significant interaction between the variables moisture and density was calculated (*P <* 0.01). Individual average weight differed significantly for the moisture (*P* < 0.01) and the density level (*P* < 0.01), wherein interactions of both variables were verified (*P* = 0.01). The highest average individual larval weight (233.3 mg FM) was obtained at 250 larvae/box in the 75% moisture group, which was ≥ 9.9% greater than in all other groups. In general, the weight of the larvae decreased successively with increasing density (*r* = −0.47; *P* = 0.05) and was positively correlated with increasing moisture level (*r* = 0.86; *P* < 0.01; Table 2). There was an 83.2% reduction in weight at 350 larvae/box reared at 70% moisture level in comparison to 75% moisture (*P* < 0.01). There was a significant difference (101.6%) within the 300 larvae/box density at different moisture levels. Within the 70% moisture treatments, the average individual larval weight was highest at a larval density of 250. Fresh and dry matter of the total harvested biomass was highest (60.0 g FM and 19.8 g DM) in the 75% moisture group at 300 larvae/box, followed by the 250 larvae/box group within the same moisture level with a mean reduction of 13.0% FM (*P* = 0.10) and 7.6% DM (*P* = 0.42), respectively. Both variables were significantly affected by the moisture (*P* < 0.01 and *P* < 0.01) and the density (*P* < 0.01), and interactions thereof were calculated (*P* < 0.01). Here, the harvested biomass improved between 35.1–108.3% FM and 35.3–132.9% DM with increasing moisture. In general, the lowest biomass was harvested at 300 larvae/box and a moisture level of 70% (Table 2). No linear relationship was determined for the density and total biomass (FM: *r* = −0.17; *P* ≥ 0.51).

**Table 2:** Growth performance and feed conversion ratio of BSF larvae reared at different moisture levels (70–75%) and varying larval densities (250–350).

	Substrate moisture (%)					
	Larval density					
Variables	70%	70%	70%	75%	75%	75%
	250 L	300 L	350 L	250 L	300 L	350 L
**Survival rate (%)**	97.9 ±3.0 ^aA^	98.0 ±2.4 ^aA^	100.0 ± 0.0 ^aA^	93.2 ± 3.0 ^aA^	96.4 ± 2.0 ^aA^	83.7 ± 4.0 ^bB^
**Average Individual larval weight (mg FM)**	158.6 ±9.0 ^aA^	105.3 ± 1.6 ^bA^	97.3 ± 4.7 ^bA^	233.3 ± 8.2 ^aB^	212.3 ± 4.2 ^aB^	178.3 ±8.1 ^bB^
**Total harvested biomass (g FM)**	39.3 ±0.5 ^aA^	28.8 ±0.4 ^bA^	32.3 ± 1.9 ^abA^	53.1 ± 1.3 ^aB^	60.0 ± 2.3 ^abB^	50.7 ± 4.7 ^acB^
**Total harvested biomass (g DM)**	13.6 ±0.2 ^aA^	8.5 ±0.1 ^bA^	9.3 ± 0.5 ^bA^	18.4 ± 0.4 ^aB^	19.8 ± 0.8 ^abB^	16.3 ± 1.5 ^acB^
**Feed conversion ratio (FCR)**	2.6 ± 0.0 ^aA^	3.5 ± 0.1 ^bA^	3.1 ± 0.2 ^bA^	1.9 ± 0.0 ^aB^	1.7 ± 0.1 ^aB^	1.6 ± 0.2 ^aB^

Data represent means ± SD (*n* = 3). Different letters within a row indicate statistically significant differences, with lowercase letters (a–c) referring to data within the moisture group and uppercase letters (A–B) referring to data within a density group (*P* < 0.05; two-way ANOVA). DM = dry matter, FM = fresh matter, FCR = feed conversion ratio

Moisture level (*P* < 0.01), density (*P* < 0.01), as well as their interactions (*P* < 0.01) significantly affected the feed conversion ratio (FCR). Regardless of the larval density, FCR improved with increasing moisture level (*P* < 0.01). The best feed conversions were obtained within the 75% moisture group (1.6–1.9), whereby no density effect was recorded (*P* ≥ 0.13). FCR was highest at 250 larvae/box and differed significantly from the 300 and 350 larvae/box groups (*P* < 0.01 and *P* < 0.01) at 70% moisture level. FCR was not found to have a linear relationship for the density (*r* = 0.20; *P* = 0.42). Given that 250 larvae/box achieved optimum results and required 16.6% less individuals, this density was considered most promising.

### Investigation of scale effects on growth performance and bioconversion

Based on the previous experiments, the optimal moisture level for a feed mixture of 50% CF and 50% SMS was determined to be 75%, yielding the highest average individual larval weight, harvested biomass and FCR. Among the tested larval densities, 250 and 300 larvae/box produced the best outcomes in terms of average individual larval weight, biomass, and FCR. The results indicated almost significant difference between the densities of 250 and 300 larvae/box at 75% moisture content (Table 2). Given that 250 larvae per box maintained optimal results despite being fewer than 300, this density was considered to be better and chosen as benchmark for the investigation of scale effects at 75% substrate moisture. Each group was provided with the same (0.4 g DM/larva) feeding rate, which was adapted to different scales between 10–2,500 g feed and 25–6,500 larvae, respectively.

The survival rate differed significantly among the scale groups (*P* < 0.01), with the 10–1,000 g feed groups achieved ≥ 92%. In the 2,500 g scale, a survival rate of 72.3% was recorded, which was 21.5−38.3% lower compared to the smaller scales (Table 3). A negative correlation between the survival rate and the scale was determined (*r* = −0.85*; P* < 0.01).

**Table 3:** Growth performance and bioconversion efficiency of BSF larvae reared at different scales (10–2,500 g DM feed provided to 25–6,500 larvae) using similar feeding rates of 0.4 g DM/larva.

	Feed provided (g DM)				
	10	50	100	1,000	2,500
Variables	25 Larvae	125 Larvae	250 Larvae	2,500 Larvae	6,500 Larvae
**Survival rate (%)**	100.0 ± 0.0 ^a^	98.7 ± 0.4 ^a^	92.0 ± 2.9 ^ab^	98.8 ± 0.5 ^a^	72.3 ± 4.8 ^b^
**Average Individual larval weight (mg FM)**	122.7 ± 3.5 ^a^	155.9 ± 2.3 ^b^	166.5 ± 2.6 ^bc^	151.7 ± 2.4 ^bd^	142.7 ± 11.4 ^ab^
**Total harvested biomass (g FM)**	2.9 ± 0.1	18.3 ± 0.3	36.4 ± 0.7	354.1 ±3.8	619.0 ±27.3
Total harvested biomass * (g FM)	*0.11 ± 0.00 ^b^	*0.15 ± 0.00 ^a^	*0.15 ± 0.00 ^a^	*0.14 ± 0.00 ^a^	*0.10 ± 0.00 ^c^
**Total harvested biomass (g DM)**	0.9 ± 0.0	6.4 ± 0.1	11.5 ±0.5	107.6 ± 7.1	159.9 ±7.9
Total harvested biomass * (g DM)	*0.04 ±0.00 ^c^	*0.05 ± 0.00 ^a^	*0.05 ± 0.00 ^ab^	*0.04 ± 0.00 ^b^	*0.02 ± 0.00 ^d^
**Feed conversion ratio (FCR)**	3.5 ± 0.1 ^b^	2.7 ± 0.0 ^c^	2.8 ± 0.1 ^c^	2.8 ± 0.0 ^c^	4.1 ± 0.2 ^a^
**Efficiency of conversion of the ingested feed (ECI)**	0.3 ± 0.0 ^ab^	0.4 ± 0.0 ^ac^	0.5 ± 0.0 ^a^	0.5 ± 0.0 ^ad^	0.5 ± 0.3 ^a^
**Frass weight (g DM)**	6.8 ± 0.2	33.4 ± 0.4	76.0 ±2.6	790.7 ± 12.8	2095.8 ±159.3
Frass weight * (g DM)	*0.27 ± 0.01^a^	*0.27 ± 0.00 ^a^	*0.30 ± 0.01 ^ab^	*0.32 ± 0.01 ^b^	*0.32 ± 0.02 ^ab^
**Substrate reduction (%)**	31.9 ± 1.9 ^a^	33.2 ± 0.7 ^a^	24.0 ± 2.6 ^ab^	20.9 ± 1.3 ^b^	16.2 ±6.4 ^ab^
**Waste reduction index (WRI; g DM/d)**	0.3 ± 0.0	1.7 ± 0.0	2.4 ± 0.3	20.9 ± 1.3	40.4 ± 15.9
Waste reduction index * (WRI; g DM/d)	*0.013 ± 0.00 ^a^	*0.013 ± 0.00 ^a^	*0.010 ± 0.00 ^ab^	*0.008 ± 0.00 ^b^	*0.006 ± 0.00 ^ab^

Data represent means ± SD (*n* = 3). Different letters (a–d) within a row indicate statistically significant differences between groups (*P* < 0.05; one-way ANOVA or Welch’s ANOVA). * = normalized data. DM = dry matter, FM = fresh matter, FCR = feed conversion ratio, ECI = efficiency of conversion of the ingested feed, WRI = waste reduction index

The highest average individual larval weight (166.5 mg FM) was obtained in the 100 g scale group and was 35.7% and 9.8% higher than in the 10 g (*P* < 0.01) and 1,000 g scales (*P* < 0.02), respectively. Contrastingly, the 2,500 g scale did not differ significantly from the 100 g scale (*P* = 0.27). The 10 g scale produced the larvae with the lowest (122.7 mg FM) average individual larval weight. No linear relationship between the average individual larval weight and scale could be calculated (*r* = −0.09; *P* = 0.73). Both fresh matter and dry matter of the total harvested biomass were significantly different among the scales (*P* < 0.01 and *P* < 0.01). Based on the normalized data, both variables were highest in the 50 g and 100 g scales with a weight of 0.15 g FM and 0.05 g DM, respectively (Table 3). The lowest normalized total biomass (0.10 g FM and 0.02 g DM) was obtained from the 2,500 g scale and was 50% (*P* < 0.01) and 150% (*P* < 0.01) lower compared to the 50–100 g scale groups, respectively. The total harvested biomass was negatively correlated with increasing scale (FM: *r* = −0.70*; P* < 0.01; DM: *r* = −0.78*; P* < 0.01). The FCR was significantly different across the treatments (*P* < 0.01). For the FCR, the smallest and largest scales had the worst FCR, while the scales between 50–1,000 had the best values ranging between 2.7–2.8. FCR correlated positively with increasing scale (*r* = 0.71*; P* < 0.01). The efficiency of conversion of the ingested feed (ECI) was lowest (0.28) in the 10 g scale and increased by 89.3% in the 1,000 g scale (*P* = 0.01). Contrastingly, the ECI was not significantly different between the 10 g and the 2,500 g scales (*P* = 0.81). Although the ECI increased successively with increasing scale, it is not linearly related to the scale. (*P =* 0.21). The normalized frass weight revealed significant differences across the scales (*P* < 0.01) and was highest (0.32 g DM) at 1,000 g and 2,500 g scales (Table 3). Interestingly, the 1,000 g scale differed significantly from the 10 g (*P <* 0.02) and 50 g scales (*P* < 0.01), whereas the 2,500 g scale did not (*P* = 0.28 and *P* = 0.24). The frass weight was found to increase with scale (*r* = 0.67*; P* < 0.01). With 33.2%, the 50 g scale group achieved the highest substrate reduction, representing a 2.1-fold higher reduction compared to the 2,500 g scale (*P* < 0.01). The substrate reduction was negatively correlated to the scale (*r* = −0.77*; P* < 0.01). The waste reduction index (WRI), larval ability in reducing the given feed, differed significantly between the treatments (*P* < 0.01). Here, the 10 g and 50 g scale groups yielded the highest WRI (0.013 g DM/d), reflecting 2.2-fold higher daily reduction values than the 2,500 g group (*P* < 0.01and *P* < 0.01). The WRI was negatively correlated with increasing scales (*r* = −0.79*; P* < 0.01).

## Discussion

### Optimization of substrate moisture

Substrate moisture is crucial in rearing BSF larvae [29, 32, 47, 48] because it interacts with substrate texture, microbial activity, and larval movements impacting larval feed consumption, in turn larval survival, development time, and prepupae size [30, 49]. Besides, moisture influences effective separation of larvae from the frass while harvesting [31]. In the current study, the survival rate showed no significant difference for the moisture levels 65, 70, and 75%. The lowest survival (~93%) was however found at 75% moisture level (S1 Table). It could be because a thin layer of water formed in this study in boxes with 75% moisture content, similar to the studies of Bekker et al. (2021) [29]. Comparably high survival rates (> 95%) were found at moisture levels of 60, 70, and 80% [28] and at 70, 75, and 80% [31]. The water holding capacity can vary for different substrates at the same moisture level. This change in water retention or release can impact larval survival and growth rate due to variations in substrate texture and the amount of free water present [33]. Our study found that SMS can be used as feed for BSF, as feeding 100% SMS or mixing it with CF poses no threat to larval survival [50]. Additionally, we observed that a 75% moisture is the upper limit; exceeding this value causes a layer of water to form on the substrate, leading to larval drowning and escape. However, larvae fed with fresh SMS could perform differently. We concur that drying and grinding the substrate is necessary to ensure a well-distributed and homogenized feed for the larvae. A significant difference in larval average individual weight was observed across moisture levels when rearing on equal amounts of CF and SMS, with weights increasing as moisture levels rose, reaching up to 243 mg FM at 75% moisture (S1 Table). A similar outcome was reported in another study using three feed types and five different moisture levels (55–75%), where the highest weight (237 mg FM) larvae were obtained at 75% on a crumbled pellet diet [33]. The larval weights observed in the current study underscore the potential of substituting CF with SMS. The lower average larval weight (115–178 mg FM) with 40–60% of SMS in Nayak et al. (2024) is due to the lower substrate moisture [50]. Thus, indicating the importance of moisture adjustment. The harvested biomass depends directly on the average individual weight of the larvae and their survival. Similar to average individual weight, the total biomass harvested from 250 larvae/box was highest at 75% moisture, increasing with higher substrate moisture (Table 1). The average individual larval weight and total biomass achieved in this study demonstrate the potential of replacing CF with SMS, with minimal impact on yield. In the current study, substrate moisture levels exceeding 75% were not tested, as it could prolong the presence of a water layer, restricting the oxygen supply required by the larvae. Consequently, it is assumed that larvae would not perform better in a 50% CF and SMS mixture with moisture levels above 75%. However, alternative feed substitutes to SMS might perform better at substrate moisture levels exceeding 75%. In this study, feed conversion ratio (FCR) was also affected by moisture, with an improved FCR of 1.9 at 75% compared to 3.8 at 65%. The higher moisture levels may have restricted O_2_ penetration into the substrate and thus increased anaerobic microbial activity, which could have led to an improvement in feed conversion. While the majority of studies did not specify the FCR, a ratio of 1.9 is among the best for various diets [19]. Substrate moisture significantly impacts BSF larvae performance, and a fixed moisture level seems to be not ideal due to variables like water-holding capacity [51]. Instead, the moisture levels should be adjusted for each substrate based on its physical properties [17]. Moisture control can also be achieved using water-absorbing ingredients like rice bran, rice husk, and coconut coir powder [52], or through aeration [31]. Active aeration was not employed in this study. However, it could be both a viable option and a necessity for larger-scale larval production in a bioreactor. With proper ventilation, BSF larvae can even thrive on substrates with up to 90% moisture content [51].

### Density optimization for the selected moisture contents

Larval density, typically measured in larvae per cm^2^ [36], is crucial when using BSF for bioconversion [38, 53]. In this study, densities of 300 and 350 larvae per 720 ml container were tested, alongside a 250 larval density for comparison, resulting in densities of 2.45, 2.85, and 2.04 larvae/cm^2^, respectively. Other studies have used densities ranging from 0.31 larvae/cm^2^ [36] to 10 larvae/cm^2^ [38, 43, 54]. These variations in larval densities complicate direct comparisons of larval performance [55]. Nevertheless, a comparison of studies is made from the available data for larval performance.

In this study, the highest density (350 larvae/box) at 75% moisture resulted in a significantly lower survival rate of 83.7% in comparison to other treatments (S2 Table). Dzepe et al. (2020) found a decreasing survival rate with increasing density for larval numbers of 1, 2, 4, 6, 8, and 10 larvae/cm^2^ [47]. It was found that average individual larval weight decreased as density increased, regardless of moisture level. However, larval weights were consistently higher at 75% moisture across all densities. The highest average individual larval weight in this study (233.3 mg FM) occurred at 75% moisture with a density of 250 larvae, while the lowest weight (97.3 mg FM) was at 70% moisture with a density of 350 larvae (Table 2). This trend has been observed in other studies as well [47, 53]. In general, differences in average individual larval weight are not only because of the density and moisture but other factors such as feed type and nutritional properties [53]. Schreven et al. (2022) concluded the same as the larval average individual weights were significantly different between the feed types (CF, CF and cameline seed press cake mix, chicken manure) [37]. Here, the larval weights decreased tremendously from 70.4 mg FM to 24.5 mg FM in chicken manure for densities of 50 and 200 larvae/container, respectively. For CF, the weight difference between the same densities was just 26.4 mg FM. Total harvested biomass in this study refers to the biomass at the end of the experiment minus the inoculated biomass. There was no linear relationship between density and total biomass (FM: *r* = −0.16, *P* = 0.51 and DM: *r* = −0.30, *P* = 0.22). The highest biomass (60.0 g FM and 19.8 g DM) was obtained for the density of 300 larvae at 75% moisture. The current results indicate that a substrate moisture level of 75% is more favorable than 70%. This adjustment provides a simple and cost-effective method to boost larval output. The increased moisture might have enhanced nutrient absorption by the larvae, contributing to the improved results. Variations in larval numbers and survival rates make it challenging to compare total harvested biomass across studies, and few have reported this metric. Gligorescu et al. (2022), for example, reported harvesting 950 and 2,000 g FM larvae per box (40 × 60 × 20 cm) at larval densities of 7 and 10 larvae/cm^2^, respectively [43]. Their study, however, involved a much larger scale, with 14,000 and 20,000 larvae per box. FCR was lowest (1.58) at 350 larvae/box at 75% substrate moisture, while it was highest (3.5) in the 300 larvae/box group at 70% moisture. Our results indicate that FCR does not have a linear correlation with density (*r* = 0.20, *P* = 0.42). However, Yakti et al. (2022) declared that the FCR values significantly differed between the densities (4.2–6.3 larvae/cm^2^) [42]. The FCR of 1.4–2.6 were measured for high protein-high fat and low protein-low fat diets, respectively [44]. The FCR values from other studies range between 2.6–4.6 [43], 3.1–4.2 [42], and 13.4 [2]. It is hence clear that the FCR is also diet dependent.

Our findings indicate that larvae fed a CF and SMS mixed diet show optimal growth at 75% substrate moisture and a density of 250 among the tested variables (S2 Table). This observation aligns with other studies; for instance, both extremely high and low densities [38, 47] and extreme moisture levels can be detrimental [34]. Higher densities increase competition but are also linked to elevated phenoloxidase levels, enhancing insect immunity [54]. Parra Paz et al. (2015) recommend larval densities between 1.2 and 5 larvae/cm^2^ [5]. The optimal combination of moisture and density depend on the substrate and should be determined through pilot tests, though lab results may not directly translate to larger scales. Considering all these, it can be summarized that the stocking density, substrate moisture, and their interactions are key factors influencing BSF larvae performance and are critical for BSF production.

### Investigation of scale effects on growth performance and bioconversion

Several authors have advocated for large-scale experiments, as results from small-scale studies cannot be directly applied to industrial settings [42]. Key reasons include shifts in environmental conditions, resource management, and operational costs [56]. The scale experiment is crucial in overcoming these challenges, as it determines the feasible number of treatments and replicates based on available time and manpower. Tasks like counting larvae, preparing feed, and harvesting are time-intensive. However, large-scale studies are essential as BSF gains popularity as an alternative animal feed. In our study, we compared scales using the same feeding rate (0.4 g DM/ larva) and climatic conditions. The smallest scale (10 g DM feed, 25 larvae) was 25 times smaller than the largest one (2,500 g DM feed, 6,500 larvae). Although the largest scale is still smaller than industrial bioreactors, the aim was to observe fundamental changes. Yang and Tomberlin (2020) compared small- and large-scale setups by rearing BSF larvae in 1.0 L and 29.5 L containers, using 307 g of feed and 614 larvae in the small scale, and 5 kg of feed with 10,000 larvae in the large scale [57]. Scaling up might alter various factors, especially in industrial settings with different rearing systems, locations, harvesting or ventilation systems [40]. Large-scale studies typically use 7 kg [40, 41] to 10 kg [42] of feed and 10,000–13,000 larvae. It is important to note that the largest scale in our study is 3–5 times smaller, yet notable differences in larval performance were observed. This is also because we have covered a broader spectrum of scales. We found significant differences in the survival rate. The lowest survival was obtained in the 2,500 g scale group (S3 Table). This is not surprising as it has been postulated that survival rate and larval growth might vary from laboratory to industrial scale [31, 42]. The survival rate was found to be 28.2% greater on the industrial scale than on a bench-top scale [57]. Yakti et al. (2022) also found that the larval mortality rate was significantly higher in small scale [42]. The contradictory result in our study could be the effect of layer of water that reduced the substrate aeration in the larger scale. The smallest scale of 10 g feed we tested was not ideal as the BSF larvae tend to live in aggregation [58]. This was not possible because of lower amount of substrate (and its depth). Miranda et al. (2020) reared 10,000 larvae in 7 kg FM feed (70% moisture) [41]. There, the average individual larval weight of larvae on the day of harvest (9 d) was between 152–170 mg FM for the four diets tested (swine, dairy and poultry manure, Gainesville diet). We observed the highest average individual weight (166.5 mg FM) in the100 g DM feed approach. In contrast, higher density (1,000 and 2,500 g DM feed) groups had a lower larval weight probably because of the less aeration within the substrate as there was a thin layer of water in the largest scale for up to three days. Moreover, we observed higher temperatures at higher densities which could be due to increased movement and interaction with conspecifics, microbial activity, varied air movements among others, which may have resulted in higher energy consumption and reduced weight gain. Similarly, the bench-top scale resulted in an average individual larval weight of 174.4 mg FM and was 24.7% higher than that of the industrial scale [57]. The total biomass obtained per larva (normalized data) decreased with upscaling (Table 3). However, the lowest scale (10 g) also had a lower total harvested biomass indicating the lower scale is not ideal even in lab-scale approaches. The FCR values in our study ranged between 2.7–4.1 (S3 Table). We found no significant difference in the FCR values for the scales 50–1,000 g feed. Gligorescu et al. (2022) state FCR values of 2.3–5.5, indicating successful and efficient production in a semi-industrial setting [43]. A lower FCR indicates better feed efficiency, meaning less feed is needed to produce a given amount of body mass. However, an FCR of 4.1 in our study is considered decent even in comparison to other small-scale studies which had much higher FCR values, namely 5.8 for municipal organic waste [20] or 10.3 for poultry and dairy manure [59]. The use of catering and household waste as BSF feed resulted in an FCR of 1.7–3.6 [60]. This highlights that the FCR, like other variables, depends on various factors such as diet composition, particle size, pH levels, moisture content, larval density, and temperature in BSF production. The efficiency of conversion of ingested feed (ECI) articulates the amount of the ingested feed converted into larval biomass. In general, the ECI increased with upscaling. The ECI parameter is not yet discussed in terms of scale in any studies. In the current study, the substrate reduction decreased with upscaling, resulting in more frass at higher scales that needs to be managed. The substrate reduction was only 16.2% in the 2,500 g scale, while it was 31.9% in the lowest scale group. Such lower substrate reduction at a larger scale can pose a challenge, particularly in industrial settings, unless the resulting frass is repurposed as a soil supplement. The waste reduction of 59.4–74.0% was measured for 10,000 larvae fed with 8 kg of a diet consisting of apple, banana, and spent grains [40]. These general differences between the studies may be because of varying fiber contents and nutrient qualities.

The scale of BSF production has recently gained attention as industrial setups expand globally [40, 43]. Our scale comparison highlights its importance for optimizing production during scaling. While bench-top experiments remain crucial for fast and economically assessing various substrates [40], results may not directly translate across different scales. This can also be seen from our results where the five different scales used led to different larval performance for the variables analyzed. The variation in larval performance may also be attributed to the vacuum packaging used during their early stages for transportation to the laboratory. While the effects of vacuum packaging on young larvae are not well understood, it could potentially have an impact. However, vacuum packaging is a standard method with which young BSF larvae are delivered from the BSF companies. Future research should integrate both small- and large-scale experiments for more comprehensive validation of results. As private companies investing in large-scale BSF production often keep their findings confidential due to competitive pressures [39], studies like ours are valuable for exploring new possibilities in mass production of BSF larvae.

In this study, we primarily conducted experiments using a 100 g DM feed mix. Returning to the initial research questions, we conclude that increasing the moisture level to 75% enhanced larval performance, as evidenced by improvements in larval weight, total biomass, and FCR. Moreover, it was observed that densities exceeding 250 larvae/box did not improve larval performance, even with substrate moisture levels above 60%. Through optimizing moisture level and larval density, we determined that a CF and SMS mix at 75% moisture and a density of 250 larvae/box yielded the best results. In the scale experiment, the highest individual larval weight (169 mg FM) and optimal FCR (2.7) were obtained with 100 g DM feed. The five laboratory scales also revealed that the scale of the experiment, even within similar feed and climate settings, resulted in differences in larval performance in terms of survival, individual larval weight, total biomass, and FCR. These results suggest that for large-scale applications, variables must be carefully controlled and minimally adjusted to replicate similar outcomes.

## Supporting information

S1 Table Optimization of substrate moisture. A comparison of three treatments with three replicates each. In the treatment column % indicates the percentage of substrate moisture and L indicates number of larvae used per treatment. Survival % = percentage of survived larvae or pupae, FM = fresh matter, DM = dry matter, FCR = feed conversion ratio.
(XLSX)

S2 Table Density optimization for selected moisture contents. A comparison of six treatments with three replicates each. In the treatment column % indicates the percentage of substrate moisture and L indicates number of larvae used per treatment. Survival % = percentage of survived larvae or pupae, FM = fresh matter, DM = dry matter, FCR = feed conversion ratio.
(XLSX)

S3 Table Investigation of scale effects on growth performance and bioconversion. A comparison of five treatments with three replicates each. In the treatment column g indicates the amount of substrate on dry matter basis and L indicates number of larvae used per treatment. Survival % = percentage of survived larvae or pupae, FM = fresh matter, DM = dry matter, FCR = feed conversion ratio, ECI = efficiency of conversion of ingested feed, WRI = waste reduction index.
(XLSX)
